# Unbiased proteomic mapping of the LINE-1 promoter using CRISPR Cas9

**DOI:** 10.1186/s13100-021-00249-9

**Published:** 2021-08-23

**Authors:** Erica M. Briggs, Paolo Mita, Xiaoji Sun, Susan Ha, Nikita Vasilyev, Zev R. Leopold, Evgeny Nudler, Jef D. Boeke, Susan K. Logan

**Affiliations:** 1 Department of Biochemistry and Molecular Pharmacology, NYU Grossman School of Medicine, Alexandria Center for Life Sciences, 450 East 29th Street, Room 321, New York, NY 10016 USA; 2 Present Address: Opentrons Labworks, Queens, NY USA; 3 Institute of Systems Genetics, NYU Grossman School of Medicine, New York, NY 10016 USA; 4 Cellarity, Cambridge, MA USA; 5 Department of Urology, NYU Grossman School of Medicine, Alexandria Center for Life Sciences, 450 East 29th Street, Room 321, New York, NY 10016 USA; 6 Howard Hughes Medical Institute, NYU Grossman School of Medicine, New York, NY USA; 7 Department of Biomedical Engineering, NYU Tandon School of Engineering, Brooklyn, NY USA

**Keywords:** LINE-1, Transcriptional regulation, Cancer, C-BERST, CRISPR Cas9 Restricted Spatial Tagging

## Abstract

**Background:**

The autonomous retroelement Long Interspersed Element-1 (LINE-1) mobilizes though a copy and paste mechanism using an RNA intermediate (retrotransposition). Throughout human evolution, around 500,000 LINE-1 sequences have accumulated in the genome. Most of these sequences belong to ancestral LINE-1 subfamilies, including L1PA2-L1PA7, and can no longer mobilize. Only a small fraction of LINE-1 sequences, approximately 80 to 100 copies belonging to the L1Hs subfamily, are complete and still capable of retrotransposition. While silenced in most cells, many questions remain regarding LINE-1 dysregulation in cancer cells.

**Results:**

Here, we optimized CRISPR Cas9 gRNAs to specifically target the regulatory sequence of the L1Hs 5’UTR promoter. We identified three gRNAs that were more specific to L1Hs, with limited binding to older LINE-1 sequences (L1PA2-L1PA7). We also adapted the C-BERST method (dCas9-APEX2 Biotinylation at genomic Elements by Restricted Spatial Tagging) to identify LINE-1 transcriptional regulators in cancer cells. Our LINE-1 C-BERST screen revealed both known and novel LINE-1 transcriptional regulators, including CTCF, YY1 and DUSP1.

**Conclusion:**

Our optimization and evaluation of gRNA specificity and application of the C-BERST method creates a tool for studying the regulatory mechanisms of LINE-1 in cancer. Further, we identified the dual specificity protein phosphatase, DUSP1, as a novel regulator of LINE-1 transcription.

**Supplementary Information:**

The online version contains supplementary material available at 10.1186/s13100-021-00249-9.

## Introduction

Long Interspersed Element-1 (LINE-1) is the only autonomous mobile element in the human genome. Over millions of years, LINE-1 sequences have accumulated in our DNA through the process of retrotransposition, entailing the copy and paste of an RNA intermediate [1, 2]. An estimated 500,000 copies of LINE-1 exist in the human genome, complicating the study of LINE-1 and its retrotransposition [3]. Yet, the majority of LINE-1 sequences are non-functional due to 5’ truncations, mutations, and inversions. These nonfunctional sequences are predominantly ancestral LINE-1 sequences, including subfamilies L1PA2-L1PA7, which can no longer mobilize [1, 4, 5]. The remaining 80–100 full length LINE-1 sequences belong to the human specific LINE-1 subfamily (L1Hs) and are capable of retrotransposition, carrying the potential to reshape our genome, alter gene expression, and disrupt genome integrity [6–8].

LINE-1 consists of a 5’ UTR, two open reading frames encoding ORF1p and ORF2p proteins, and a 3’UTR with a polyA tail [9, 10]. A fully downstream sense promoter is located within the 5’UTR, controlling LINE-1 mRNA expression [11]. Additionally, an antisense promoter has been identified within the 5’UTR that has been shown to control expression of a third open reading frame on the antisense strand, ORF0, as well as alternative antisense transcript expression [12–14]. Throughout the evolution of LINE-1 sequences, the 5’UTR has acquired new regulatory sequences, exhibiting a rapid evolution of host factor binding, especially KRAB zinc finger binding proteins [15]. LINE-1 encoded proteins, ORF1p and ORF2, have remained relatively conserved and both are instrumental in retrotransposition [16, 17]. ORF1p is a nucleic acid chaperone that forms homotrimers and binds LINE-1 mRNA [18, 19]. ORF1p has also been shown to bind ssDNA as well as non-LINE-1 mRNAs [20, 21]. ORF2p’s endonuclease and reverse transcriptase domains provide the enzymatic activity necessary for retrotransposition [22, 23]. Once expressed, ORF1p and ORF2p bind LINE-1 mRNA forming the ribonucleoprotein (RNP). Upon nuclear breakdown during mitosis, the RNP enters the nucleus where the ORF2p endonuclease nicks the DNA and creates a new copy of LINE-1 through target-primed reverse transcription (TPRT) [24–27]. LINE-1 has also been shown to retrotranspose in non-dividing cells, suggesting an additional mode of entry into the nucleus [28].

Many mechanisms have evolved to silence LINE-1 expression in somatic cells, limiting its potential to mobilize. DNA methylation, histone modifications, RNA interference, and transcription factor binding have all been shown to play a role in limiting LINE-1 expression and restricting retrotransposition [29–36]. Many of these mechanisms are disrupted in cancer cells, allowing for the re-expression and mobilization of LINE-1 [37–39]. LINE-1 protein ORF1p has been observed in approximately 47% of tumors, and has been a proposed indicator of aggressive disease in some cancers [37, 40, 41]. New LINE-1 insertions have also been detected in around 53% of tumors studied. In prostate cancer, 60% of tumors contained at least one new LINE-1 insertion, and the rate of retrotransposition was accelerated in metastatic disease [38]. However, this varies among cancers because in clear cell renal cell carcinoma (ccRCC), no new insertions were detected in tumor samples assessed [42]. This variation in LINE-1 expression and retrotransposition between types of cancers suggests possible cell-type specific regulation of LINE-1. While LINE-1 insertions are frequently found in introns and non-coding regions, new exonic insertions have also been detected in tumor suppressor genes, including *APC* in colorectal cancer [33, 43]. In addition to directly disrupting a gene, new insertions can alter the regulatory landscape of the genome by inducing new patterns of methylation [44–46].

The potential for LINE-1 to alter gene expression and drive genomic instability suggests that it may promote cancer. Better understanding of LINE-1 transcriptional regulation will provide insight into its dysregulation and activity in cancer cells. To date, Suv39h H3K9me3, the HUSH complex, SETDB1, and KAP1 were all shown to regulate LINE-1 elements in embryonic cells [47–50]. Additional studies have identified Myc, CTCF, YY1 and RUNX3 binding sites on the LINE-1 5’UTR through motif analysis [32, 51–53]. Further functional analysis of these transcription factors have revealed roles in transcriptional regulation and transcription initiation [32, 52]. Here, we have optimized a unique CRISPR C-BERST model to conduct an unbiased study identifying transcriptional regulators of active LINE-1 (L1Hs) in cancer cells. While this technique can identify traditional transcription factors, it can also identify proteins that are not directly bound to DNA but also play role in transcriptional regulation.

## Results

### Targeting the LINE-1 5’UTR promoter with dCas9 C-BERST

**Fig. 1:**
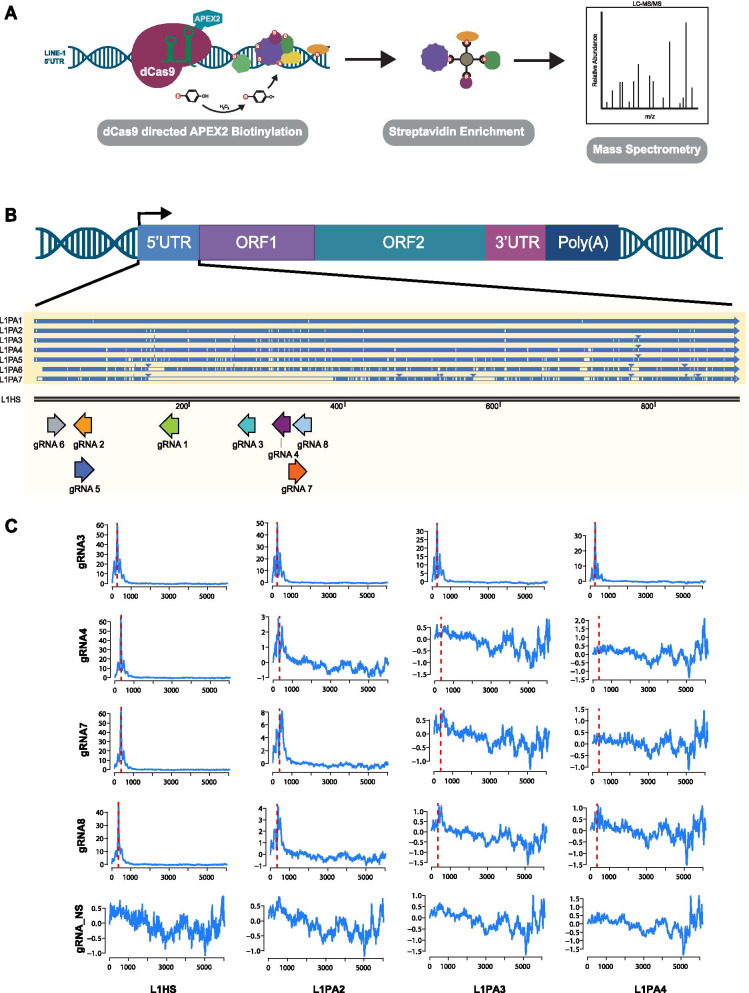
Utilizing the C-BERST method to target LINE-1 5’UTR. **A** An APEX2 tagged dCas9 is recruited to the LINE-1 5’UTR promoter using L1Hs specific gRNAs. Cells are incubated with biotin-phenol for 30 min and treated with hydrogen peroxide for 1 min, triggering the biotinylation of proteins located at the LINE-1 5’UTR promoter. Biotinylated proteins are enriched through streptavidin immunoprecipitation and identified through mass spectrometry. Figure and method based on Gao et al. [54]. **B** Alignment of L1PA2-L1PA7 to L1Hs. Blue lines designate correct alignment to L1Hs, cream lines designate mismatch alignment to L1Hs, and arrowheads mark additional misaligned sequence missing from L1Hs. Eight guide RNAs (gRNA) were designed to target the 5’UTR of LINE-1. Preference went to guides that aligned well with L1Hs, but lacked alignment to older LINE-1 sequences (L1PA2-L1PA7). **C** Chromatin immunoprecipitation (ChIP) of dCas9 was performed in cells expressing each of the eight LINE-1 5’UTR gRNAs, as well as a non-targeting control (NS). ChIP reads were aligned to LINE-1 L1Hs, as well as older L1PA2-L1PA7, using MapRRCon analysis software [32]. Red line denotes the target location of each gRNA. Representative data shown for gRNA 3, 4, 7 and NS. Complete data set for all gRNAs can be found in Supplemental Figure 1

To better understand LINE-1 transcriptional regulation in cancer cells, we utilized the dCas9 C-BERST (d**C**as9–APEX2 **B**iotinylation at genomic **E**lements by **R**estricted **S**patial **T**agging) method to map regulatory proteins bound to the LINE-1 promoter [54]. C-BERST utilizes a nuclease deficient Cas9 (dCas9) fused to the ascorbate peroxidase APEX2. When expressed, dCas9-APEX2 is directed to specific DNA loci using guide RNAs (gRNA). Upon treatment with biotin-phenol and hydrogen peroxide, APEX2 generates biotin-phenoxyl radicals which covalently biotin-label proteins within an ~ 20 nm radius (Fig. 1A) [54]. The LINE-1 promoter is located within its 5’UTR and is transcribed as part of LINE-1 mRNA to preserve it during retrotransposition [11]. To map regulatory proteins directing LINE-1 transcription in cancer cells we directed gRNAs to the LINE-1 5’UTR promoter region.

To selectively direct dCas9 to active, retrotransposition competent LINE-1 sequences, we designed eight gRNAs that specifically targeted the L1Hs 5’UTR. gRNA design targeted regions of the 5’UTR that aligned poorly to older LINE-1 sequences, L1PA2-L1PA7 (Fig. 1B). Next, we conducted chromatin immunoprecipitation (ChIP) of dCas9 in the presence of each 5’UTR gRNAs and one non-targeting gRNA control (NS). Using the LINE-1 specific software MapRRCon [32], we aligned Cas9 ChIP reads to L1Hs, as well as to the older LINE-1 elements, L1PA2-L1PA7. As anticipated, all eight gRNAs aligned to the targeted region in the 5’UTR of L1Hs (Supplementary Figure S1). When we aligned ChIP reads to the older, nonfunctional LINE-1 sequences (L1PA2-L1PA7), gRNA 4, gRNA 7, and gRNA 8, were enriched for L1Hs binding and began to lose alignment quality to older sequences beginning with L1PA7. Guides 4, 7 and 8 had lost almost all alignment to L1PA3-7, making them the least likely to target ancestral LINE-1 (Fig. 1C). All other guides showed alignment with L1PA2 and L1PA3, as shown with gRNA 3, but lost significant peaks in the 5’UTR as they were aligned to older LINE-1 sequences (Fig. 1C, Supplementary Figure S1). gRNA 4 and gRNA 7 were chosen for the C-BERST assay due to their high level of specificity for younger, active, L1Hs sequences, and minimal recruitment to the older L1PA2-L1PA7 sequences.

### Identification of LINE-1 5’UTR localized proteins through dCAS9-APEX2 biotinylation

**Fig. 2:**
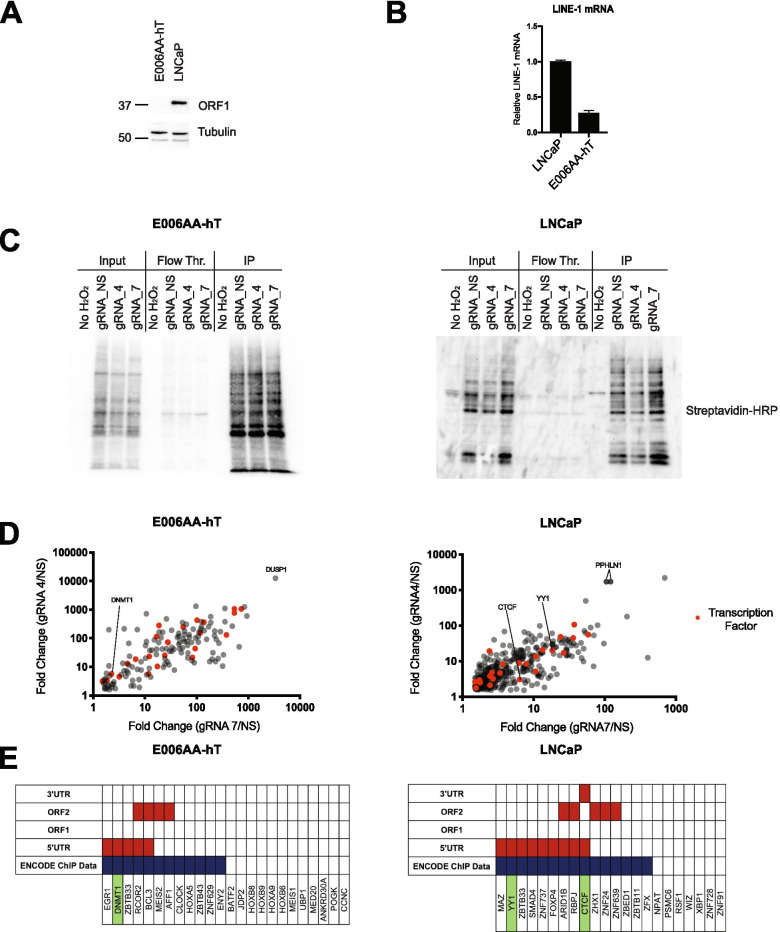
C-BERST identification of 5’UTR biotinylated transcription factors. **A** Western blot of LINE-1 ORF1p expression in whole cell lysates of E006AA-hT and LNCaP cells. (Tubulin loading control). **B** Relative LINE-1 mRNA levels as assessed by qPCR. **C** Western blot of C-BERST streptavidin immunoprecipitation. Cells expressing gRNA-4, gRNA-7, or gRNA-NS were harvested, and biotinylated proteins were collected by streptavidin immunoprecipitation. A no hydrogen peroxide control (No H_2_O_2_) was included in the harvest to eliminate endogenously biotinylated proteins. Input, flow through, and immunoprecipitation (IP) were blotted with streptavidin HRP. **D** Proteins identified with C-BERST that were enriched at least 1.5 × in both LINE-1 targeting gRNAs (gRNA-4, gRNA-7) when compared to the non-targeting control (gRNA NS). Transcription factors have been highlighted in red. **E** Complete list of transcription factors enriched at least 1.5 × in both LINE-1 targeting gRNAs (gRNA-4, gRNA-7) when compared to non-targeting control (gRNA NS). Proteins previously identified to regulate LINE-1 are highlighted in green. Blue squares denote ENCODE ChIP data availability. Red squares denote peak on specified LINE-1 location by MapRRCon analysis

The two cell lines we used to identify LINE-1 5’UTR bound proteins were LNCaP and E006AA-hT. LNCaP cells are an androgen dependent prostate cancer cell line that has been shown to express LINE-1 ORF1 protein and mRNA (Fig. 2A and B). E006AA-hT cells express no detectable LINE-1 ORF1 protein [55], and have very low levels of LINE-1 mRNA (Fig. 2A and B). While first thought to be a prostate cancer cell line, E006AA-hT cells were later found to be a clone of renal cell carcinoma cell line 786‐O [56]. The variation of LINE-1 expression in these cell lines provides a compelling model to better understand LINE-1 transcriptional activation and repression in cancer cells. For example, proteins bound to the LINE-1 5’UTR in E006AA-hT cells may have suppressive activity since the cells do not express LINE-1.

Each cell line was stably transfected with dCas9-APEX2 and a gRNA (gRNA 4, gRNA 7, or gRNA NS). As previously optimized, cells were sorted to select for low dCas9 (mCherry) expression in order to reduce background [54]. dCas9-APEX2 was induced with doxycycline treatment for 21 h, cells were incubated with biotin-phenol for 30 min, and treated with hydrogen peroxide for 1 min. After quenching the hydrogen peroxide and isolating nuclei, biotinylated proteins were collected through streptavidin immunoprecipitation and proteins were identified by mass spectroscopy (Fig. 2C and D). Our screen revealed 22 transcription factors in LNCaP cells (356 total enriched proteins), and 24 transcription factors in E006AA-hT cells (149 total enriched proteins), that were enriched at least 1.5 × above gRNA NS in both LINE-1 specific guides (gRNA 4, gRNA 7) (Fig. 2D and E). In both screens, we identified proteins previously shown to regulate LINE-1 expression, including YY1, PPHLN1, and CTCF in LNCaP cells, and DNMT1 in E006AA-hT cells (Fig. 2E) [32, 48, 52, 53, 57].

### Validating transcription factors with MapRRCon

**Fig. 3:**
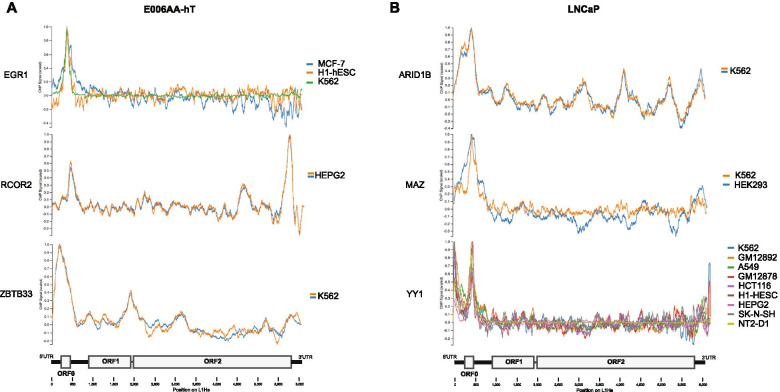
MapRRCon ChIP plots of C-BERST identified proteins. MapRRCon analysis of C-BERST enriched proteins in **A** E006AA-hT and **B** LNCaP cells. Each plot is an analysis of available ENCODE ChIP data, in multiple cell lines (listed on the right side of each plot). Position of observed peaks is noted by the full length L1Hs diagram along the bottom of the figure. Additional MapRRCon plots can be found in Supplemental Figure 2

To assess the presence of enriched transcription factors on the LINE-1 5’UTR we analyzed available ENCODE ChIP data with MapRRCon software. For LNCaP cells, we analyzed 15 transcription factors from our C-BERST screen that had available ENCODE ChIP data and found that 12 of these proteins (80%) showed peaks on LINE-1 with 9 mapping to the 5’UTR (60%) (Figs. 2E and 3B). Similarly, we also analyzed 12 transcription factors enriched in the E006AA-hT C-BERST assay and found that 7 (58.3%) contained peaks on LINE-1 with 5 mapping to the 5’UTR (41.6%) (Figs. 2E and 3A). In both cell lines there were transcription factors that showed more than one peak on full length LINE-1, including RCOR2 (Fig. 3). Interestingly, ENCODE ChIP data was collected from multiple cell types and was analyzed by MapRRCon for peaks on LINE-1. Many of the identified transcription factors had peaks in multiple cell types, suggesting a broad role in regulating LINE-1 transcription (Fig. 3), while others, such as BCL3, showed clear differences between cell types (Supplemental Figure S2).

### C-BERST reveals novel regulators of LINE-1 mRNA expression

**Fig. 4:**
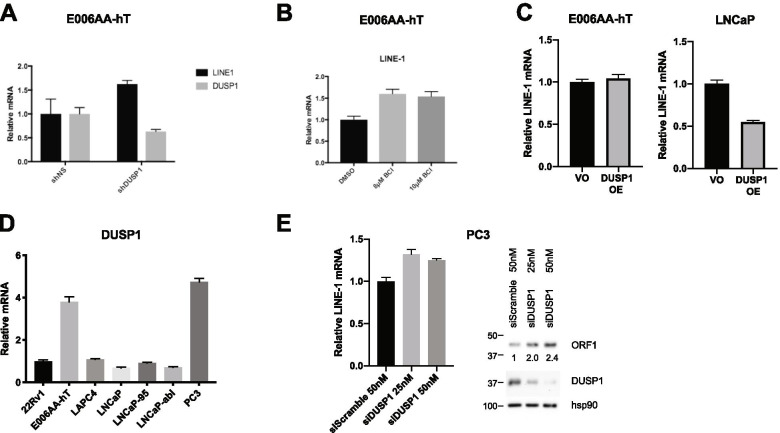
DUSP1 modulates LINE-1 mRNA expression. **A** qPCR of LINE-1 mRNA levels in E006AA-hT cells expressing shRNA to DUSP1 or non-targeting control (NS). **B** qPCR of relative LINE-1 levels in E006AA-hT cells that were treated with vehicle (DMSO) or DUSP1 inhibitor BCI. **C** qPCR of relative LINE-1 mRNA levels in E006AA-hT or LNCaP cells overexpressing vector only (VO) or DUSP1. **D** qPCR of DUSP1 mRNA levels in panel of prostate cancer cell lines (including E006AA-hT renal cell carcinoma line). **E** qPCR of relative LINE-1 mRNA levels (left) and western blots of LINE-1 ORF1p, DUSP1, and hsp90 (loading control) protein levels (right) in PC3 cells that were treated with siScramble or siDUSP1 siRNA. ORF1 protein levels relative to hsp90 were quantified, siScramble was set to 1

The top enriched protein in our E006AA-hT C-BERST screen was DUSP1, a dual specificity protein phosphatase known to inhibit MAPK [58]. In order to test the effect of DUSP1 on LINE-1 expression, we conducted a knockdown of DUSP1 with shRNA in E0006AA-hT cells. We evaluated LINE-1 mRNA levels by qPCR and found a 1.6 fold increase of LINE-1 transcript levels upon DUSP1 knockdown (Fig. 4A). Next, we used a DUSP1 inhibitor, BCI, and assessed its effect on LINE-1 transcript levels by qPCR. Again, we saw a 1.5–1.6 fold increase in LINE-1 transcript levels upon treatment (Fig. 4B). DUSP1 was only found in the E006AA-hT C-BERST screen, suggesting that it is inhibiting LINE-1 expression; to test this hypothesis, we examined its effect on LINE-1 transcript levels in LNCaP cells. Upon over expression of DUSP1 in LNCaP cells, we observed a 45% decrease in LINE-1 transcript levels (Fig. 4C) supporting our hypothesis. Overexpression of DUSP1 in E006AA-hT cells resulted in no change in LINE-1 levels, likely due to DUSP1 saturation in these cells. We also observed high DUSP1 mRNA levels in PC3 cells, a prostate cancer cell line shown to have low levels of LINE-1 expression (Fig. 4D) [55]. To examine whether DUSP1 was playing a role in regulating LINE-1 in PC3 cells, we knocked down DUSP1 with siRNA and evaluated LINE-1 mRNA and ORF1 protein levels (Fig. 4E). After knockdown, we observed an increase in both LINE-1 mRNA and ORF1p protein levels. ORF1p protein levels increased by 2.0 and 2.4 fold compared to siScramble. Together, our results show DUSP1 plays a role in suppression of LINE-1 transcription in cancer cells.

## Discussion

The dysregulation of LINE-1 in cancers and its potential to perturb genomic integrity underscores the importance of understanding LINE-1 transcriptional regulation. However, the abundance of ancestral LINE-1 sequences in the human genome compounds the challenge of studying this repetitive element. Here, we utilized chromatin immunoprecipitation and LINE-1 specific analysis software, MapRRCon, to mitigate this challenge and assess the L1Hs specificity of eight CRISPR Cas9 gRNAs. Our analysis revealed three gRNAs, spanning a 47 bp section of the 5’UTR, that showed high specificity to L1Hs and low recruitment to L1PA2-L1PA7. These three gRNAs differed from the L1PA2 consensus sequence by 1 (gRNA4), or 2 (gRNA7, gRNA8) base pairs, and differed from L1PA3 by 2 (gRNA4, gRNA8) or 3 (gRNA7) base pairs. While these differences between our gRNAs and older LINE-1 sequences were subtle, our ChIP analysis shows that they disrupted gRNA recruitment to L1PA2-L1PA7. Out of these three gRNAs, we chose to use gRNA 4 and gRNA7 for our C-BERST assay. While gRNA 8 also showed high specificity to L1Hs, it did not fully align with an active L1Hs sequence that we previously identified to be expressed in LNCaP cells (Xp22.2(2)) [21]. Since Xp22.2(2) was one of the highest LINE-1 loci expressed in LNCaP cells, we decided to exclude it as a guide. However, gRNA 8 may be used as a viable LINE-1 gRNA in alternative cell types. The analysis of these gRNAs not only set up a platform for targeting L1Hs with C-BERST, it also provides insight for additional CRISPR applications used to target LINE-1 L1Hs.

We preformed C-BERST in two different cell types, LNCaP and E006AA-hT, in order to assess different mechanisms of LINE-1 regulation. In LNCaPs, we have previously shown that many different LINE-1 loci are being actively expressed, with notable ORF1p levels observed [21, 55]. While LNCaP cells express a substantial number of LINE-1 loci, they contained a range of expression from highly expressed LINE-1 loci to non-detectable loci as analyzed by ORF1p RNA immunoprecipitation [21]. On the other hand, E006AA-hT cells have very low LINE-1 mRNA expression with no detectable ORF1p expression (Fig. 2A and B). In LNCaP cells we identified 356 proteins that were enriched at the 5’UTR in both gRNAs, 22 of which were transcription factors. In E006AA-hT cells, we only observed 149 enriched proteins, including 24 transcription factors. The discrepancy in total number of enriched proteins in each cell line may be due to the variation in LINE-1 expression in LNCaP cells. Different LINE-1 loci may have a variety of protein complexes recruited for either LINE-1 activation or repression, yielding a higher number of enriched proteins. This discrepancy may also be cell type specific, or possibly due to higher background in LNCaP cells. Among the transcription factors identified only one, ZBTB33, was enriched in both LNCaP and E006AA-hT cell lines. These results suggest a dramatic difference in LINE-1 regulation between cell and/or cancer type.

The C-BERST method was developed to identify proteins bound or localized to specific loci. Our screen revealed a number of proteins that have previously been shown to regulate LINE-1 expression, as well as identified new putative LINE-1 regulators. Transcription factors YY1 and CTCF were both enriched at the 5’UTR in LNCaP cells and have previously been shown to bind to the LINE-1 5’UTR and modulate transcription [32, 52, 53]. PPHLN1, a component of the HUSH complex, was highly enriched in our LNCaP screen, and has also been shown to inhibit LINE-1 expression [48, 59]. The HUSH complex maintains H3K9me3 through the recruitment of SETDB1, another protein shown to regulate LINE-1 expression and found to be enriched in our assay [60, 61]. However, other than PPHLN1, no other components of the HUSH complex were identified as enriched, perhaps because LINE-1 is not repressed at many LINE-1 loci in LNCaP cells. Additionally, DNMT1, a DNA methyltransferase that has previously been shown to repress young LINE-1 elements, was enriched at the 5’UTR in E006AA-hT cells [57]. In addition to these previously identified proteins, our MapRRCon analysis identified five proteins in E006AA-hT cells and nine in LNCaP cells that had confirmed ChIP peaks in the LINE-1 5’UTR. Additional proteins were shown to have peaks in ORF2 and in the 3’UTR in our MapRRCon analysis. It is possible that these proteins were enriched due to secondary chromatin structure at the LINE-1 loci, however, further analysis is needed to determine their potential role in LINE-1 regulation.

Dual Specificity Phosphatase 1, DUSP1, was the most highly enriched protein in E005AA-hT cells. DUSP1 inactivates MAPK through dephosphorylation of threonine/tyrosine, including p38 MAPK, JNKs and ERKs [58, 62]. We speculate that DUSP1 may dephosphorylate MAPKs and thereby alter downstream transcription factor activity in protein complexes regulating the L1HS 5’UTR. In early prostate and bladder cancers, DUSP1 is expressed at higher levels, however, as histological grade progresses, DUSP1 levels decrease. Our results show that DUSP1 consistently contributes to LINE-1 repression in E006AA-hT cells and PC3 cells. Interestingly, high DUSP1 expression was observed in cell lines with lower LINE-1 expression (Fig. 4D) [55]. While our C-BERST results strongly suggest it is localized around the 5’UTR, further analysis is needed to explore DUSP1 substrates instrumental in LINE-1 regulation. Since we required proteins to be enriched in both guides, it is possible that our stringency eliminated important cofactors and DUSP1 substrates. Our application of C-BERST enabled us to identify proteins both directly bound to the LINE-1 5’UTR (ZBTB33, ERG1, GATAD1 etc.), as well as transient regulators that may have been missed in traditional sequence and ChIP analysis (DUSP1). Overall, our LINE-1 optimized C-BERST assay enables the identification of cell type specific LINE-1 transcriptional regulators.

## Conclusions

The abundance of ancestral LINE-1 sequences in the genome presents a significant challenge to studying active L1Hs regulation. In our study, we identified three CRISPR Cas9 gRNAs that specifically target active L1Hs, with minimal binding to older LINE-1 sequences. We also utilized these gRNAs in the restricted spatial tagging method, C-BERST, to identify proteins localized to the LINE-1 5’UTR promoter. Our application of the C-BERST method identified both known and novel LINE-1 transcriptional regulators, including the dual specificity phosphatase, DUSP1, in cancer cells. Our optimization of the C-BERST method to specifically target the L1Hs promoter has created a tool that can be used to better understand the regulation of LINE-1 expression in cancer cells.

## Materials and methods

### Cell culture

E006AA-hT (CRL-3277) and LNCaP (CRL-1740) cell lines were purchased from the ATCC. E006AA-hT cells were maintained in DMEM supplemented with 10% FBS. LNCaP cells were maintained in RPMI 1640 supplemented with 10% FBS. Cells were assessed regularly for mycoplasma contamination.

### siRNA knockdown

Human DUSP1 siRNA SMARTpool (#L-003484–02-005) and non-targeting control pool (#D-001810–10-05) was purchased from Dharmacon. PC3 cells (2.5 × 10^5^ cells per well) were seeded on 6 well plates. PC3 cells were transfected with 25 and 50 nM siRNAs using Lipofectamine RNAiMAX reagent (Life Technologies) according to the manufacturer’s instructions. Transfections were performed on two consecutive days. RNA and protein were collected 72 h after the first transfection. Whole cell lysates were harvested in RIPA buffer (50 mM Tris pH 8, 150 mM NaCl, 1% NP-40, 0.1% SDS, 10 mM EDTA, 10 μg/mL aprotonin and leuptin, 1 mM PMSF, and 1 mM Na_3_VO_4_) and protein concentration was quantified using a Bradford Assay.

### shRNA knockdown

DUSP1 and scramble shRNA were cloned into a pTRIPZ backbone. pTRIPZ plasmid and viral packaging plasmids were transfected into HEK 293 T cells with Lipofectamine Reagent (Thermo Fisher 18324012) (2 μg pMD2G, 3 μg psPAX2, and 5 μg pTRIPZ) and virus was collected and filtered after 48 h. Viral supernatant (4 mL) was supplemented with fresh media (2 mL) and Polybrene (8 μg/mL) and incubated with E006AA-hT cells for 4 h. After 48 h, cells were treated with puromycin (1 μg/mL). Once selected, shRNA expression was induced with 1 μg/mL doxycycline for 48 h.

### sgRNA creation and design

sgRNAs were designed using the MIT guide RNA design tool (CRISPR.MIT.edu). sgRNAs with at least one mismatch to older LINE-1 (L1PA2-L1PA7) sequences were prioritized. Once sequences were selected, sgRNAs constructs were cloned by into the pEJS614_pTetR-P2A-BFPnls/sgNS by replacing sgNS sequence with LINE-1 targeting guide sequence. Guide sequences can be found in Supplementary Table 2.

### DUSP1 overexpression

DUSP1 sequence was amplified from a DUSP1 GenScript plasmid (OHu10841D) and cloned into a pCW57-MCS1-2A-MCS2 backbone (Addgene #71782). Overexpression plasmid and viral packaging plasmids were transfected into HEK 293 T cells with Lipofectamine Reagent (Thermo Fisher 18324012) (2 μg VSV-G, 3 μg gag-pol, and 5 μg Overexpression plasmid) and virus was collected and filtered after 48 h. Viral supernatant (4 mL) was supplemented with fresh media (2 mL) and Polybrene (8 μg/mL) and incubated with E006AA-hT or LNCaP cells for 4 h. After 48 h, cells were treated with puromycin (1 μg/mL). Once selected, DUSP1 expression was induced with 1 μg/mL doxycycline for 48 h.

### BCI treatment

E006AA-hT cells were seeded in a 6-well plate and incubated overnight at 37 °C. Cells were then treated with DMSO or BCI (Axon Medchem #2178) for 3 h at 37 °C. RNA was collected with the Qiagen RNeasy Plus Mini Kit (74134) as described below and assessed by qPCR.

### RNA isolation and qPCR

RNA was isolated from cells using the Qiagen RNeasy Plus Mini Kit (74134) and contaminating DNA was digested using a Turbo DNA-free DNase digestion (Thermo Fisher Scientific AM1907) according to manufacturer’s protocol. cDNA was made using the Verso cDNA kit (Thermo Scientific- AB1453A). qPCR was conducted using SYBR Green Master Mix (Life Technologies 4344463) and relative mRNA levels were calculated using ΔΔCT. RPL19 was used as an internal control for normalization. Primer sequences can be found in Supplementary Table 2. qPCR LINE-1 primers have been previously published [63].

### Chromatin immunoprecipitation

E006AA-hT and LNCaP cells (~ 20 × 10^6^) stably expressing dSpyCas9-mCherry-APEX2 and gRNA were grown to 80% confluency. Cells were treated with 250 nM Sheild1 (Clontech) and 2 μg/mL doxycycline for 21 h to induce dCas9 expression. Cells were crosslinked with formaldehyde (1% formaldehyde in PBS) at room temperature for 10 min, and quenched with 1 mL of 2.5 M glycine, gently shaking for 5 min. Cells were washed with PBS and pelleted at 425xg for 5 min at 4 °C and resuspended in Farnham lysis buffer (5 mM PIPES pH 8.0, 85 mM KCL, 0.5% NP-40, Halt protease inhibitor (Thermo Fisher-87786)). Suspension was re-pelleted and flash frozen in liquid nitrogen. Pellets were resuspended in 1 mL Farnham lysis buffer with Halt protease inhibitor, passed through a 25 gauge syringe 15 times, and spun at 425xg for 5 min at 4 °C. Pellets were resuspended in RIPA buffer (1 × PBS, 1%NP-40, 0.5% Na-deoxycholate, 0.1% SDS, Halt protease inhibitor) and passed through a 25 gauge syringe 20 times. Lysates were sonicated in a Diagenode Bioruptor for 30 min, 30 s on, 30 s off at 2 °C and spun at 20800xg for 10 min. Sheared DNA was incubated with 4 μg mCherry antibody (Thermo PA5-34,974) overnight at 4 °C and incubated with 50μL protein A/G beads for 3 h at 4 °C. Beads were washed 5 × with LiCl wash buffer (100 mM Tris pH 7.5, 500 mM LiCl, 1% NP-40, 1% Na-deoxycholate) for 3 min, and once with TE buffer (10 mM tris–HCl pH7.5, 0.1 mM Na_2_EDTA) for 1 min. Beads were resuspended in Proteinase K/SDS solution (0.5% SDS, 0.2 mg/mL Proteinase K, 1X TE) and incubated at 55 °C for 3 h and 65 °C overnight to reverse crosslinks. Samples were placed on magnetic strip to collect supernatant. 600μL of PB and 4μL of RNaseA (17500u/mL) were added to the samples, and samples were purified using the Qiagen PCR purification kit (28104). Sample was eluted twice with 35μL of 10 mM Tris pH 8. Illumina libraries were generated using the NEB Next DNA Library Prep Ultra II kit (E7645S) according to manufacturer’s protocol. Libraries were sequenced on an Illumina NextSeq 500. Reads were demultiplexed with Illumina bcl2fastq v2.20 requiring a perfect match to indexing BC sequences.

### Cell sorting

LNCaP and E006AA-hT cells were treated with doxycycline (2 μg/mL) and Sheild1 (250 nM) for 21 h prior to sorting. Cells were sorted using the SONY SY3200 parallel sorter (SONY Biotechnology, San Jose, CA), using a 100 µm orifice nozzle and system pressure of approximately 25 psi. Double positive cells for mCherry and BFP were purified as previously described [54].

### C-BERST assay

Biotinylation: Seven 15 cm plates of E006AA-hT or LNCaP cells (~ 6 × 10^7^) expressing a gRNA and dCas9-APEX2 were treated with 2 μg/mL doxycycline and 250 nM Sheild 1 for 21 h. Cells were incubated for 30 min with biotin-phenol (500 μM) at 37 °C, and 1 mM H_2_O_2_ was then added to cells for 1 min at room temperature. To stop the biotinylation reaction, quencher solution (5 mM trolox, 10 mM sodium ascorbate, and 10 mM sodium azide) was added and cells were placed on ice. Three additional washes with quencher solution were performed, followed by two washes with PBS.

Nuclear Isolation: Cells were scraped from plates and centrifuged at 300 × g for 5 min at 4 °C. Pellet was resuspended in 7.5 nuclei isolation buffer (10 mM PIPES pH 7.4, 0.1% NP-40, 10 mM KCl, 2 mM MgCl_2_, 1 mM DTT and Halt protease inhibitor). Cells were incubated on ice for 10 min and ruptured using a Dounce homogenizer (~ 20x). Cells were further incubated on ice for 20 min and homogenization was repeated. Lysate was gently added to a sucrose cushion that contained 20 mL of 30% sucrose and 3.5 mL 10% sucrose (10 mM PIPES pH 7.4, 10 mM KCl, 2 mM MgCl_2_, 30% or 10% sucrose, and 1 mM DTT). Sucrose cushion and cell lysate was spun at 1000 × g for 15 min. Supernatant was removed and nuclei (pellet) was resuspended in 800μL PBS. Suspension was spun at 1500 × g for 5 min at 4 °C. 500μL RIPA lysis buffer (50 mM Tris–HCl pH 7.5, 150 mM NaCl, 0.125% SDS, 0.125% sodium deoxycholate, 1% Triton X-100) was added and samples were incubated at 4 °C. Lysates were sonicated in a Diagenode Bioruptor for 15 min (30 s on, 30 s off), and centrifuged for 10 min at 15,800 × g, 4 °C. Protein concentrations were measured using a Bradford Assay and samples were normalized.

Immunoprecipitation: MyOne Streptavidin T1 Dynabeads (Thermo Fisher 65,601) (400μL) were added to each sample and incubated at 4 °C overnight. Beads were washed with RIPA (twice), 1 M KCl, 0.1 M Na_2_CO_3_, 2 M urea in 10 mM Tris–HCl pH 8.0, and again with RIPA (twice). After washes, beads were processed for mass spectroscopy (see On-beads digestion of streptavidin-bound proteins). Protocol was based off of the previously described C-BERST technique [54].

### Western blot/streptavidin blot

Cell lysates were boiled at 98 °C for 5 min in SDS loading buffer. Samples were resolved by SDS-PAGE on polyacrylamide gels, and transferred to PVDF using the BioRad Trans-Blot Turbo Transfer System. Blots were blocked in 5% BSA in TBS and probed with streptavidin-HRP (Thermo Fisher SA10001), ORF1p (Millipore MABC1152), DUSP1 (Cell Signaling 48625), or HSP90 (BD Biosciences 610419). Western blots were developed using BioRad Clarity Western ECL Substrate (1705060S) and visualized on an iBrightCL1000 Imager. Protein bands were quantified using ImageJ [64].

### On-beads digestion of streptavidin-bound proteins

Streptavidin beads were washed twice with 1 mL 50 mM NH_4_HCO_3_ to exchange the buffer. Washed beads were then resuspended in 50 μl 50 mM NH_4_HCO_3_ containing 20 ng/μl trypsin/Lys-C (Promega) followed by overnight incubation at 37 °C with vigorous mixing in a thermoshaker (Eppendorf). After incubation beads were pelleted and supernatants were transferred to new tubes. Samples were acidified by adding 5 μl 20% heptafluorobutyric acid, incubated at room temperature for 5 min and clarified by 5-min centrifugation at 16000 g. Peptides from clarified samples were desalted using C18 spin tips (Thermo Scientific) according to manufacturer’s instructions. Desalted peptides were dried under vacuum and redissolved in 0.1% formic acid prior to LC–MS analysis. Peptide concentration was measured at 205 nm on Nanodrop One (Thermo Scientific).

### LC–MS analysis

Peptides were analyzed by LC–MS on Orbitrap Fusion Lumos mass spectrometer coupled with Dionex Ultimate 3000 UHPLC. During each run, 0.5–2 μg of peptides from individual samples were injected and resolved on 50-cm long EASY-Spray column (Thermo Scientific) by 90-min long linear gradient of 4–40% acetonitrile in 0.1% formic acids at flowrate 0.25 μl/min. The method for data-dependent acquisition was based on published protocol [65] with exception that each cycle was set to last for 2 s instead of 3 s.

Peptides identification and label-free quantitation was done in the Proteome Discoverer 2.1. The protein database for Sequest HT search engine included human proteome downloaded from UniProt (www.uniprot.org) and amino acid sequence of streptavidin from Streptomyces avidinii. Parameters were set to search for peptides of at least 5 amino acids long, containing at most 2 missed trypsin cleavages. Dynamic modifications were set to include: phosphorylation of serine, threonine or tyrosine, acetylation of protein N-terminus, mono- and dimethylation of lysine and arginine. MS1-based label-free quantitation was done using the “Precursor Ions Area Detector” module in Proteome Discoverer. Samples were first normalized by intensity of streptavidin detected. Samples with an area value of 0 were replaced with the lowest MS1 intensity detected. Next, H_2_O_2_ only (endogenous biotinylation) values were subtracted from the + H_2_O_2_ samples (gRNA NS, gRNA 4 and gRNA7). Mean was calculated from replicates and targeted guides (gRNA 4 and gRNA 7) were divided by gRNA NS values for each protein detected. Proteins with at least 1.5 × greater than gRNA NS control were considered enriched and included in further analysis.

## Supplementary Information

**Additional file 1: Supplemental Figure 1.** Complete ChIP assessment for CRISPR Cas9 gRNA localization to LINE-1 gRNA1-8 and gRNA-NS were expressed with dCas9 in cells. ChIP was performed with each guide and ChIP data was assessed with MapRRCon to quantify LINE-1 (L1Hs, L1PA2-L1PA7) localization of each gRNA. Position on full length LINE-1 along X-axis. Fold enrichment above input along Y-axis. Red dotted line marks gRNA target site.

**Additional file 2: Supplemental Figure 2.** Additional MapRRCon ChIP Plots of C-BERST Enriched Transcription Factors. ChIP plots of enriched transcription factors with identified LINE-1 peaks.

**Additional file 3: Supplemental Table 1.** C-BERST Enriched proteins in LNCaP and E006AA-hT cells. Complete list of proteins that were enriched at least 1.5 fold in gRNA 4 and gRNA 7 when compared to the non-targeting control (gRNA NS).

**Additional file 4: Supplemental Table 2.** gRNA and qPCR Primer Sequences

## Data Availability

Reagents will be available upon request.

## Abbreviations

LINE-1: Long Interspersed Element-1
C-BERST: DCas9-APEX2 Biotinylation at genomic Elements by Restricted Spatial Tagging
DUSP1: Dual specificity protein phosphatase 1
