# Supplementary figures and images for: Unbiased proteomic mapping of the LINE-1 promoter using CRISPR Cas9
Source: Mob DNA. 2021 Aug 23;12:21. doi: 10.1186/s13100-021-00249-9 (PMC8381588; doi:10.1186/s13100-021-00249-9)

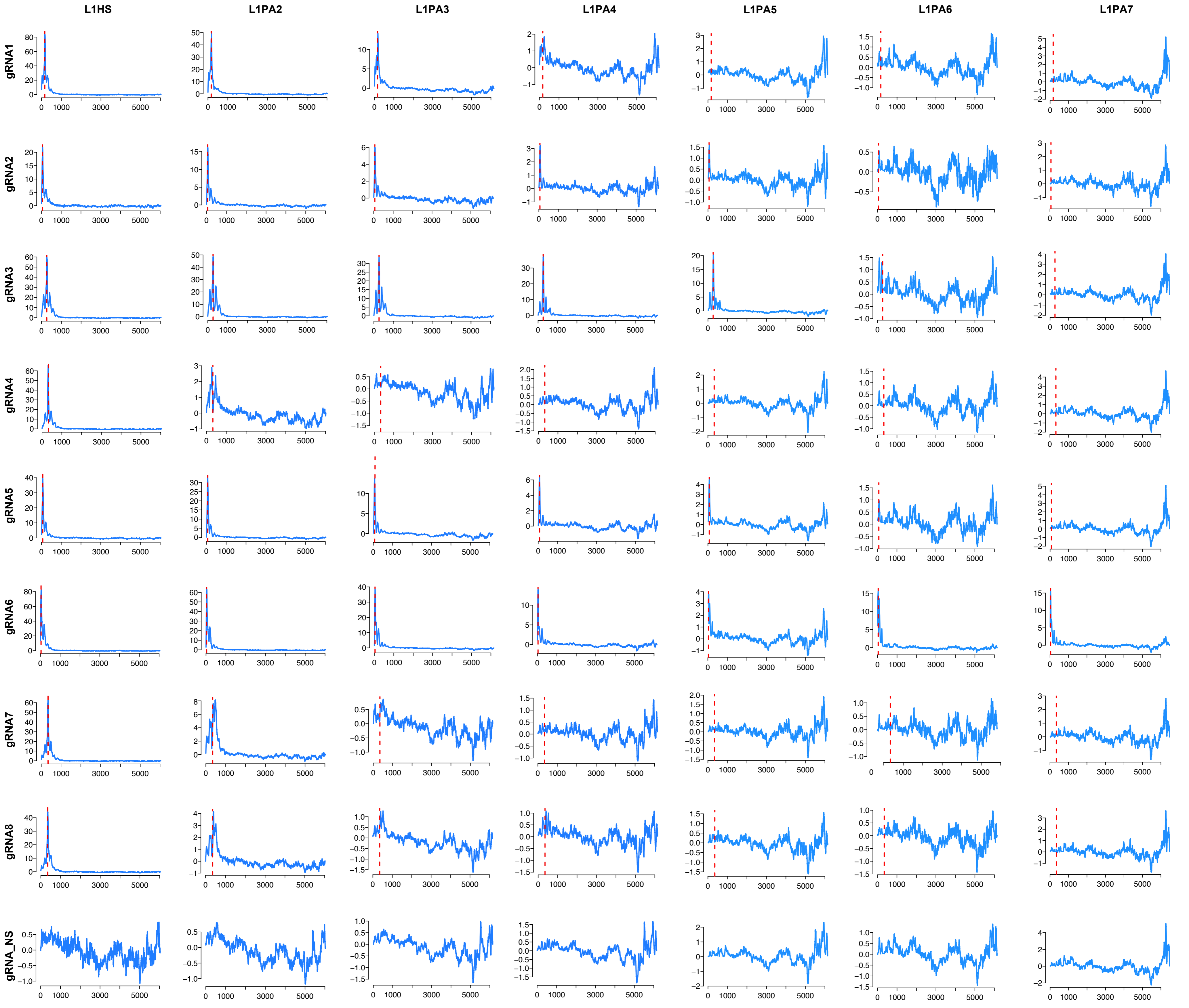

Supplement: Supplementary file 1 — Additional file 1: Supplemental Figure 1. Complete ChIP assessment for CRISPR Cas9 gRNA localization to LINE-1 gRNA1-8 and gRNA-NS were expressed with dCas9 in cells. ChIP was performed with each guide and ChIP data was assessed with MapRRCon to quantify LINE-1 (L1Hs, L1PA2-L1PA7) localization of each gRNA. Position on full length LINE-1 along X-axis. Fold enrichment above input along Y-axis. Red dotted line marks gRNA target site. [file 13100_2021_249_MOESM1_ESM.pdf]

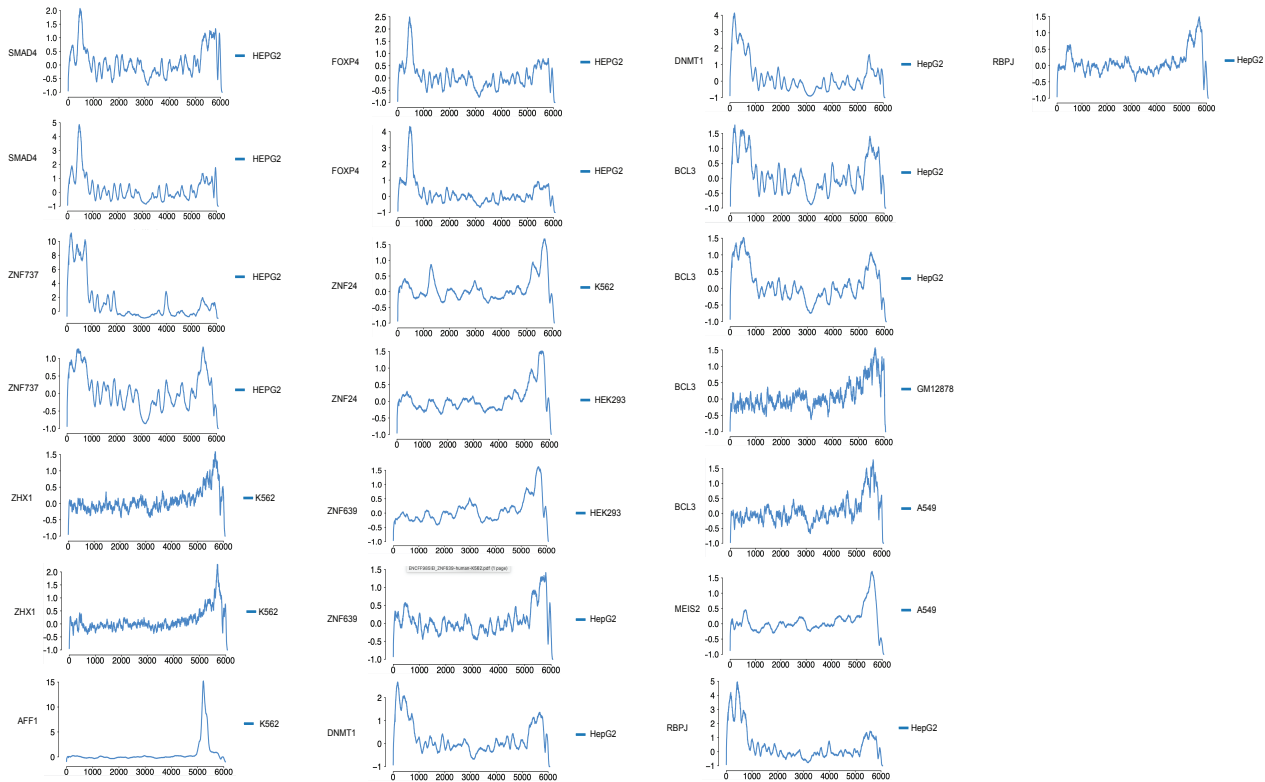

Supplement: Supplementary file 2 — Additional file 2: Supplemental Figure 2. Additional MapRRCon ChIP Plots of C-BERST Enriched Transcription Factors. ChIP plots of enriched transcription factors with identified LINE-1 peaks. [file 13100_2021_249_MOESM2_ESM.pdf]
